# Urocortin Neuropeptide Levels Are Impaired in the PBMCs of Overweight Children

**DOI:** 10.3390/nu14030429

**Published:** 2022-01-18

**Authors:** Sina Kavalakatt, Abdelkrim Khadir, Shihab Kochumon, Dhanya Madhu, Sriraman Devarajan, Maha Hammad, Nada Alam-Eldin, Samia Warsame, Hessa Al-Kandari, Maria AlMahdi, Rasheed Ahmad, Heikki A. Koistinen, Jaakko Tuomilehto, Fahd Al-Mulla, Jehad Abubaker, Ali Tiss

**Affiliations:** 1 Biochemistry and Molecular Biology Department, Research Division, Dasman Diabetes Institute, Kuwait City 15462, Kuwait; sina.kavalakatt@dasmaninstitute.org (S.K.); abdelkrim.khadir@dasmaninstitute.org (A.K.); dhanya.madhu@dasmaninstitute.org (D.M.); maha.hammad@dasmaninstitute.org (M.H.); nada.alamaldin@dasmaninstitute.org (N.A.-E.); jehad.abubakr@dasmaninstitute.org (J.A.); 2 Faculty of Medicine, University of Helsinki, 00014 Helsinki, Finland; 3 Immunology and Microbiology Department, Research Division, Dasman Diabetes Institute, Kuwait City 15462, Kuwait; Shihab.Kochumon@dasmaninstitute.org (S.K.); rasheed.ahmad@dasmaninstitute.org (R.A.); 4 Research Division, Dasman Diabetes Institute, Kuwait City 15462, Kuwait; sriraman.devarajan@dasmaninstitute.org (S.D.); samia.warsame@dasmaninstitute.org (S.W.); fahd.almulla@dasmaninstitute.org (F.A.-M.); 5 Medical Division, Dasman Diabetes Institute, Kuwait City 15462, Kuwait; Hessa.AlKandari@dasmaninstitute.org (H.A.-K.); maria.almahdi@dasmaninstitute.org (M.A.); 6 Department of Medicine, University of Helsinki and Helsinki University Hospital, 00014 Helsinki, Finland; heikki.koistinen@helsinki.fi; 7 Minerva Foundation Institute for Medical Research, 00290 Helsinki, Finland; 8 Department of Public Health and Welfare, Finnish Institute for Health and Welfare, 00271 Helsinki, Finland; tuomilehto@hotmail.com; 9 Department of Public Health, University of Helsinki, 00014 Helsinki, Finland; 10 Diabetes Research Group, King Abdulaziz University, Jeddah 21589, Saudi Arabia

**Keywords:** urocortin, UCN3, CRH, obesity, children

## Abstract

The corticotropin-releasing hormone (CRH) and urocortins (UCNs) have been implicated in energy homeostasis and the cellular stress response. However, the expression of these neuropeptides in children remains unclear. Therefore, we determined the impact of obesity on their expression in 40 children who were normal weight, overweight, and had obesity. Peripheral blood mononuclear cells (PBMCs) and plasma were used to assess the expression of neuropeptides. THP1 cells were treated with 25 mM glucose and 200 µM palmitate, and gene expression was measured by real-time polymerase chain reaction (RT-PCR). Transcript levels of neuropeptides were decreased in PBMCs from children with increased body mass index as indicated by a significant decrease in UCN1, UCN3, and CRH mRNA in overweight and obese children. UCN3 mRNA expression was strongly correlated with UCN1, UCN2, and CRH. Exposure of THP1 cells to palmitate or a combination of high glucose and palmitate for 24 h increased CRH, UCN2, and UCN3 mRNA expression with concomitant increased levels of inflammatory and endoplasmic reticulum stress markers, suggesting a crosstalk between these neuropeptides and the cellular stress response. The differential impairment of the transcript levels of CRH and UCNs in PBMCs from overweight and obese children highlights their involvement in obesity-related metabolic and cellular stress.

## 1. Introduction

Profound changes in lifestyle such as increased food intake and reduced physical exercise have led to alarming increases in childhood obesity, thus raising public health concern worldwide [1]. Obesity is involved in various metabolic impairments, including insulin resistance, dyslipidemia, and hormonal imbalances, which might impact children’s growth and induce long-lasting health consequences [1,2].

Imbalance in hormonal regulation and energy homeostasis resulting from obesity induces insulin resistance and low-grade chronic inflammation. Activation of the cellular stress response system reflects an attempt to maintain energy balance by the brain and peripheral organs [2,3]. The corticotropin-releasing factor (CRF) family of neuropeptides, consisting of CRH, urocortins (UCN)-1, 2, and 3), and their receptors (CRHR1 and CRHR2) play an important role in the regulation of energy balance and food intake as well as behavioral and endocrine responses in mammals [4,5]. They are concomitantly expressed in the brain and peripheral organs, such as the heart, pancreas, skeletal muscle, and adipose tissue [6,7,8] suggest multiple sites of interaction of central neurochemistry circuitry.

UCN1, 2, and 3 exhibit cardioprotective effects [9,10] and are able to modulate feeding behavior by reducing food intake and body weight gain in rodents [11]. Although UCN3 overexpression protects against high-fat diet (HFD)-induced metabolic dysregulation [6] and increased energy expenditure [12], decreased food intake and insulin sensitivity were observed in UCN3-null rodents [13]. In contrast, UCN2 knockout mice display insulin sensitivity and resistance against HFD-related effects [14]. Furthermore, altered expression of UCN1, CRH, and UCN2 occur during inflammation-induced irritable bowel disease [15]. We and others have also reported impaired UCN3 expression associated with obesity, type 2 diabetes (T2D), and polycystic ovary syndrome [8,16,17]. Moreover, because of the co-localization of UCN3 with quantitative trait loci for obesity, UCN3 and CRFR2 were suggested as targets for obesity treatment [18].

Spexin is another neuropeptide involved in energy homeostasis and was shown to decrease food intake following injection into HFD-induced obese rodents or goldfish [19,20]. Spexin levels in the context of obesity remain controversial. One study reported decreased levels in both adults and children with obesity and diabetes [21], whereas another showed no difference in serum spexin levels between normal weight, obese, and obese with diabetes adolescents [22]. We recently reported decreased plasma spexin levels in obese adults, which were normalized because of regular, moderate physical exercise [23].

We have recently reported the dysregulated plasma profile of UCNs, CRH, and spexin plasma levels in overweight and obese children compared with normal weight controls (In press, Obesity, 2021). Here, using representative children from different body mass index (BMI) groups, we determined the effect of obesity on the expression of these neuropeptides in PBMCs as a surrogate cell type commonly used for gene expression profiling studies [24,25].

## 2. Materials and Methods

### 2.1. Study Population Characteristics and Anthropometric Measurements

The study population consisted of 40 children (8 normal weight, 10 overweight, and 22 obese) with a mean age of 12 years as detailed in Table 1. The study was approved by the Dasman Diabetes Institute’s Review Board (study approval number RA-2011-018, 15 December 2013) and conducted according to principles of the Declaration of Helsinki. Before enrollment; written informed consent was obtained from all the children’s guardians. Children between 6 and 17 years of age were enrolled. The exclusion criteria were any chronic or endocrine disease.

Anthropometric measurements were obtained according to the World Health Organization recommendations through a standard physical examination performed by a pediatric endocrinologist. Children wore light clothes and no footwear during body weight measurement, and percent body fat was calculated using a Pediatric Body Composition Analyzer/Segmental (GAIA KIKO Jawon Medical, Gyeongsan, South Korea). A stadiometer was used to measure height and BMI percentile was calculated according to the Centers for Disease Control and Prevention (CDC) growth chart. Body mass index (BMI) was used to classify the subjects as normal weight (≥5th to ≤85th percentile), overweight (>85th to ≤95th percentile), or obese (>95th percentile) [26]. No nutritional or diet data were collected.

### 2.2. Blood Sample Collection and Analysis

Following overnight fasting, venous blood was collected into ethylenediamine tetraacetic acid (EDTA) tubes. Sterile Ficoll-Hypaque density gradient medium tubes were used to separate peripheral blood mononuclear cells (PBMCs), and plasma was collected, aliquoted, and stored at −80 °C. A Siemens Dimension RXL chemistry analyzer (Diamond Diagnostics, Holliston, MA, USA) was used to measure lipid and glucose profiles. A variant device (Biorad, Hercules, CA, USA) was utilized for hemoglobin A1c (HbA1c) measurements. The assessment of insulin and liver function enzymes was performed with Access2 and AU480 Systems (Beckman Coulter, Brea, CA, USA), respectively. The HOMA-1R (homeostatic model assessment of insulin resistance index) was calculated with the formula:

$$\text{HOMA-IR}=\frac{\left(\mathrm{glucose}\;\times \;\mathrm{insulin}\right)}{22.5}$$

ELISA kits were used to measure circulating plasma levels of CRH (#LS-F5352, Lifespan Biosciences, Seattle, WA, USA), UCN1 (#LS-F6155, Lifespan Biosciences, Seattle, WA, USA), UCN2 (#LS-F39013, Lifespan Biosciences, Seattle, WA, USA), UCN3 (#LS-F12902, Lifespan Biosciences, Seattle, WA, USA), spexin (#EK-023-81, Phoenix Pharmaceuticals, Burlingame, CA, USA), insulin, and ultrasensitive C-peptide (Mercodia AB, Uppsala, Sweden). Sample dilutions were ascertained through optimization and ELISAs were performed according to the manufacturer’s instructions. The absorbances were measured with a Synergy H4 plate reader (BioTek, Winooski, VT, USA). A Bio-plex 200 system was used to measure selected obesity and diabetes markers using the following array panels: (Metabolism/Obesity 5-Plex Human ProcartaPlex™ Panel 1 (EPX09A-15804-901), Metabolism/Obesity 9-Plex Human ProcartaPlex™ Panel 2 (EPX09A-15804-901), and MILLIPLEX MAP Human Diabetes Panel, Premixed 5 Plex Assay, (HDIAB-34K-PMX5)). Quantification and fluorescence measurements were performed using Bio-plex manager software v6 (BioRad, Hercules, CA, USA).

### 2.3. Cell Culture and Treatment

The human monocytic leukemia cell line, THP-1, was purchased from the American Type Culture Collection and grown in RPMI-1640 culture medium (Gibco, Life Technologies, Grand Island, NY, USA) supplemented with 10% fetal bovine serum (Gibco, Life Technologies, Grand Island, NY, USA), 2 mM glutamine (Gibco, Invitrogen, Grand Island, NY, USA), 1 mM sodium pyruvate, 10 mM HEPES, 0.05 mM β-mercaptoethanol, 50 U/mL penicillin, and 50 μg/mL streptomycin (P/S; (Gibco, Invitrogen, Grand Island, NY, USA), and incubated at 37 °C (with humidity) in 5% CO_2_.

THP1 cells were seeded at a density of 1 million cells/mL in a 12-well plate followed by treatment with high glucose at 25 mM (HG), palmitic acid at 200 µM (PA) or a combination of HG (25 mM) and PA (200 µM) for 4 and 24 h, respectively. D-Mannitol (20 mM) was added as an osmotic control. After treatment, the cells were harvested for RNA isolation and RT-PCR gene expression analysis.

### 2.4. RNA Extraction and Gene Expression by Real-Time Quantitative PCR

RNA extraction from PBMCs and THP1 cells was performed using Trizol reagent. Extracted RNA was converted to cDNA using the High-capacity cDNA Reverse Transcription kit (Applied Biosystems, Foster City, CA, USA). Real-time PCR was performed using SYBR green reagent (Qiagen, Valencia, CA, USA) for custom primers and Taqman gene expression assays on a Rotorgene Q (Qiagen, Germantown, MD, USA) and Applied biosystem 7500(Applied Biosystems, Foster City, CA, USA) respectively. Ct values were determined from the respective instrument software. For normalization, *GAPDH* was used as a housekeeping gene. Relative gene expression was calculated using the ΔΔCt method. The primers used are listed in Supplementary Table S1.

### 2.5. Protein Extraction and Analysis by Western Blotting

Western blots were performed using THP1 cells. Whole cell protein extracts were prepared using radioimmunoprecipitation assay (RIPA) buffer as reported earlier [17]. Protein concentrations were quantified using Bradford method and samples were prepared in sample loading buffer. Proteins were resolved on 10% sodium dodecyl sulfate polyacrylamide gel electrophoresis (SDS-PAGE) gels, transferred onto polyvinylidene fluoride (PVDF) membranes and probed with anti-UCN1 (PA5-75189, Invitrogen, CA, USA), anti-UCN2 (LS-C-335681-200, Lifespan Biosciences, Seattle, WA, USA), anti-UCN3 (bs-2786R, Bioss antibodies, Woburn, MA, USA), anti- CRF (ab184238, Abcam, Cambridge, UK), anti-SPEXIN (LS-C73416, Lifespan Biosciences, Seattle, WA, USA) and anti-GAPDH (ab2302, Millipore, Burlington, MA, USA) overnight at 4 °C. Following washes and incubation with rabbit horseradish peroxidase-conjugated secondary antibody, proteins were detected using West Femto ECL reagent (Thermo Scientific, Waltham, MA, USA). Protein bands were captured using Versadoc 5000 system (Bio-Rad, Hercules, CA, USA) and GAPDH was used as an internal loading control.

### 2.6. Statistical Analysis

Statistical analyses were performed with SPSS software version 25.0 (IBM SPSS Statistics for Windows, IBM Corp. Armonk, NY, USA). For continuous variables, descriptive statistics were used to report the mean and standard deviation. One-way ANOVA with Bonferroni’s post hoc test was used to evaluate the differences between BMI groups. Correlations between variables were assessed with a Spearman’s test. A one-way ANOVA with repeated measurements followed by Sidak´s post hoc test was used to calculate *p* values between treatment groups in cell lines from 3 independent experiments. *p* < 0.05 was considered statistically significant.

## 3. Results

### 3.1. Study Population Characteristics

The study population consisted of 40 children (*n* = 8, 10, and 22 representing normal weight, overweight, and obesity, respectively) with a mean age of 12 years. Their physical, clinical, and metabolic characteristics are summarized in Table 1.

Overweight children had a significantly higher body fat percentage (*p* = 0.003). They were insulin resistant, as reflected by elevated insulin concentrations and HOMA-IR, and they had significantly diminished glucagon levels compared with normal weight children (*p* < 0.05). Despite not being statistically significant, glucose levels were higher in this group. Obesity markers (leptin, neutrophil gelatinase-associated lipocalin (NGAL), retinol-binding protein 4 (RBP4), soluble intercellular adhesion molecule-1 (sICAM-1), and zinc alpha 2-glycoprotein (ZAG)). were increased in the overweight group compared with the normal weight control group (Table 1). Although circulating plasma levels of UCN1 exhibited an insignificant decrease in overweight children, circulating UCN2 and UCN3 levels were significantly elevated (*p* < 0.05). Obese children had an impaired lipid profile as evidenced by a significant decrease in HDL levels (*p* = 0.013) compared with the normal weight controls. Insulin and HOMA-IR levels were also significantly higher in obese children (*p* < 0.05). Whereas leptin and TNFα displayed increased trends, C-peptide and glucagon were significantly decreased in the obese group (*p* < 0.05). However, no significant changes were observed in the circulating levels of UCN1, UCN2, UCN3, CRH, or spexin in the obese group compared with the normal weight group (Table 1). When comparing the overweight and obese groups, plasma levels of CRH, UCN2, and UCN3 were decreased in the latter group, whereas spexin levels were increased (*p* < 0.05).

### 3.2. Effects of Bodyweight on Transcript Levels of CRF Peptides in PBMCs

**Figure 1:**
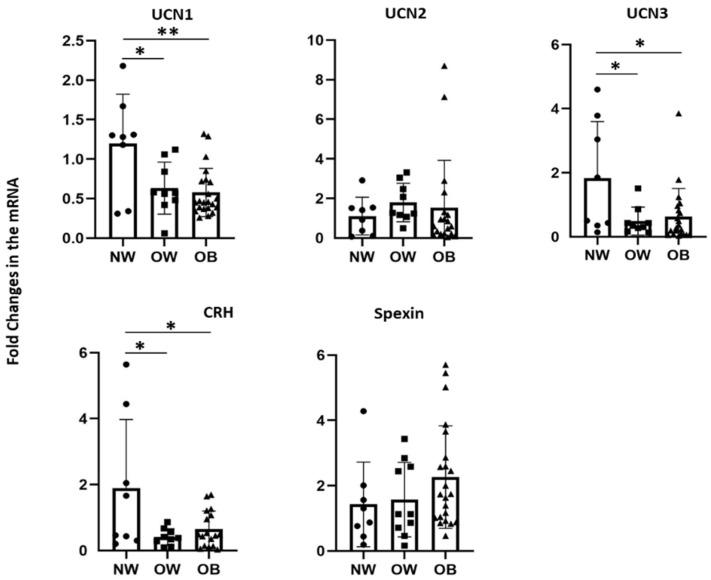
Gene expression levels in PBMCs according to body weight. mRNA expression levels of UCN1, UCN2, UCN3, CRH, and spexin were measured by quantitative real-time PCR in PBMCs from normal weight (*n* = 8), overweight (*n* = 10), and obese children (*n* = 22). Data are normalized to internal GAPDH and presented as fold-changes compared with normal weight data. One-way ANOVA followed by Bonferroni´s post hoc test were used to determine the significance of difference in means between groups (* *p* < 0.05, ** *p* < 0.01). NW (•), OW (■), and OB (▲).

To determine the role of specific neuropeptides in obesity, we measured their mRNA expression in PBMCs from children representing the three groups (Figure 1). The results indicated a significantly decreased level of UCN1, UCN3, and CRH transcripts in both overweight and obese children compared with the normal weight group. UCN2 levels showed more individual variation between the three groups, which did not allow us to draw clear trends between them. Similarly, spexin mRNA levels exhibited an increased trend with increased body weight without reaching statistical significance (Figure 1).

Spearman’s rank correlation analysis was performed to evaluate the association of transcript levels of UCN 1, 2, 3, CRH, and spexin with various clinical parameters as well as with each other (Table 2). Overall, significant negative correlations were observed for UCN1 expression and obesity markers (BMI, percentile, body fat percentage, and cholesterol), for UCN3 and TNFα and NGAL, as well as for CRH and TNFα, RBP4, ZAG, and circulating UCN2 levels (*p* < 0.05). UCN2 and UCN3 expression levels, however, correlated positively with circulating UCN3 levels (Table 2). In addition, the transcript levels of CRH, UCN1, UCN2, and UCN3 exhibited a strong positive correlation with one another (Table 2).

### 3.3. Metabolic Stress Affects the Expression of UCN1,2,3, CRH, and Spexin in THP1 Cells

**Figure 2:**
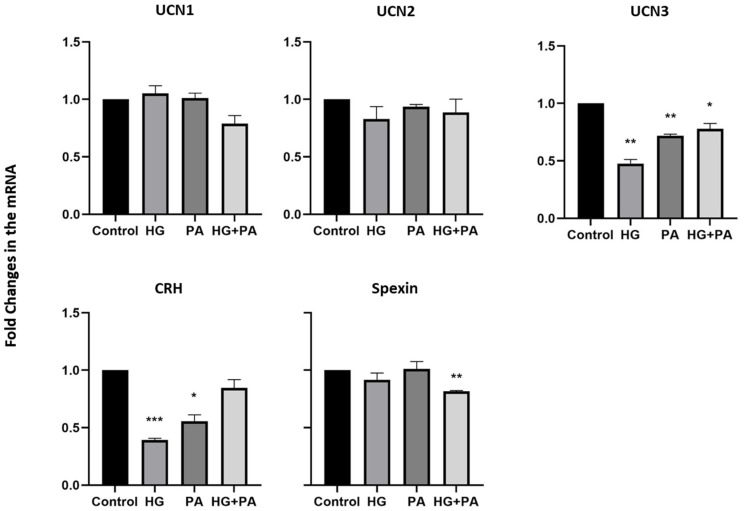
Gene expression in THP1 cells treated for four hours. mRNA expression levels of UCN1, UCN2, UCN3, CRH, and spexin were measured by quantitative real-time PCR in THP1 cells treated with high glucose (25 mM), palmitate (200 µM) or the combination for 4 h (*n* = 3 for each condition). Data are normalized to internal GAPDH and presented as fold-change compared with control data. One-way ANOVA with repeated measurements followed by Sidak´s post hoc test were used to determine the significance of the differences in means between the groups (* *p* < 0.05, ** *p* < 0.01, *** *p* < 0.001).

**Figure 3:**
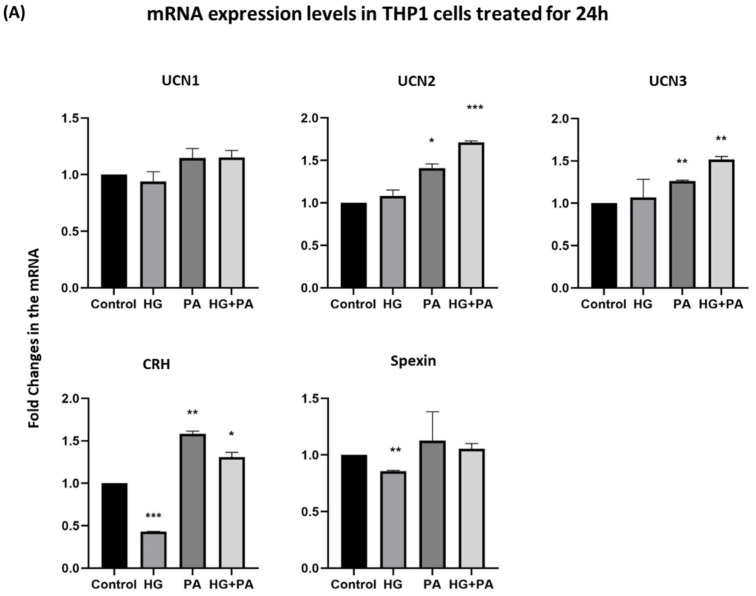

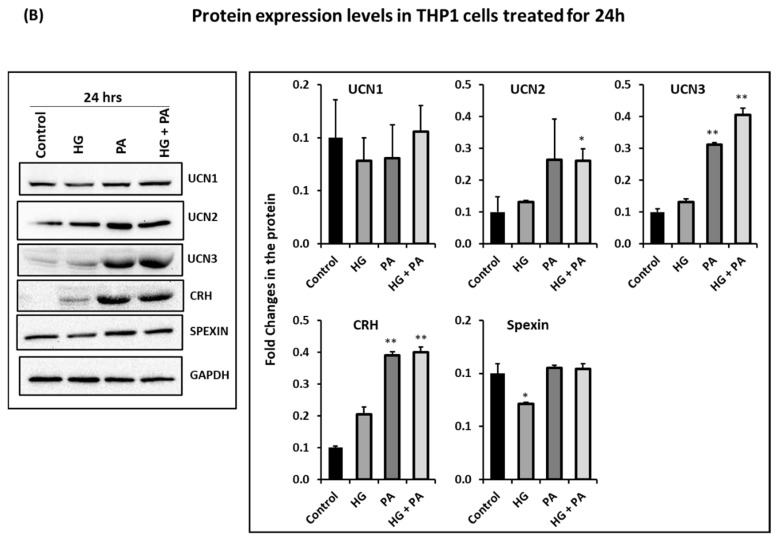
Gene and protein expression levels in THP1 cells treated for twenty-four hours. mRNA (**A**) and protein (**B**) expression levels of UCN1, UCN2, UCN3, CRH, and Spexin were respectively measured by quantitative real-time PCR and Western blots in THP1 cells treated with high glucose (25 mM), palmitate (200 µM) and the combination for 24 h (*n* = 3 for each condition). Data are normalized to internal GAPDH and presented as fold-change compared with control data. One-way ANOVA with repeated measurements followed by Sidak´s post hoc test were used to determine the significance of the differences in means between the groups (* *p* < 0.05, ** *p* < 0.01, *** *p* < 0.001).

To elucidate the role of the studied neuropeptides in obesity, THP1 human monocyte cells were treated with HG (25 mM), palmitate (PA) (200 µM), or a combination of both. When THP1 cells were treated for a short time (4 h), a significant decrease in UCN3 and CRH mRNA levels was observed under most treatment conditions (Figure 2). The effects of these treatments were, however, limited on the other neuropeptides (UCN1, UCN2, and spexin). When treated for a longer time (24 h), THP1 cells exhibited a marked increase in the mRNA levels of UCN2, UCN3, and CRH in the presence of PA or the combination of HG and PA (Figure 3A). Exposure to HG resulted in a decrease in CRH and spexin mRNA levels. Under similar experimental conditions and after 24h treatment, we extracted proteins from THP1 and assessed their expression using Western blotting. Protein levels of these neuropeptides displayed comparable trends to those obtained in the mRNA levels (Figure 3B and Supplementary Figure S1).

**Figure 4:**
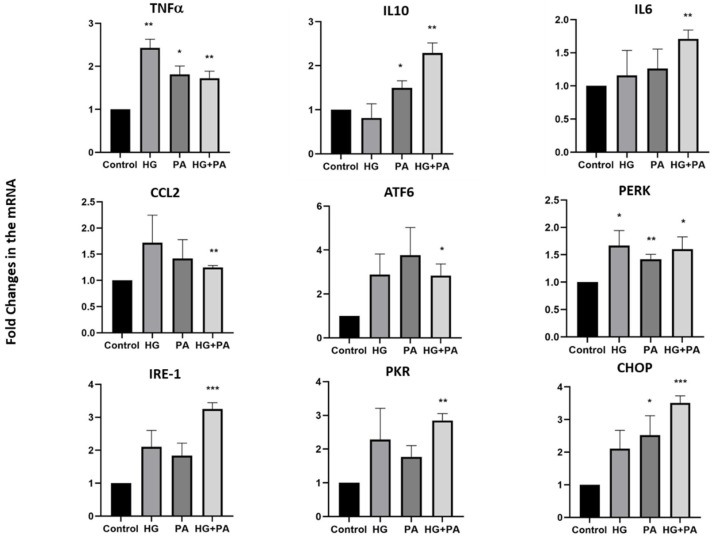
Gene expression levels of inflammatory and ER stress markers in THP1 cells treated for four hours. Gene expression levels of inflammatory markers (TNFα, IL10, IL6, CCL2) and ER stress markers (ATF6, PERK, IRE1, PKR and CHOP) were measured by quantitative real-time PCR in THP1 cells treated with high glucose (25 mM), palmitate (200 µM) and the combination for 4 h (*n* = 3 for each condition). Data are normalized to internal GAPDH and presented as fold-change compared with Control data. One-way ANOVA with repeated measurements followed by Sidak´s post hoc test were used to determine the significance of differences in means between the groups (* *p* < 0.05, ** *p* < 0.01, *** *p* < 0.001).

**Figure 5:**
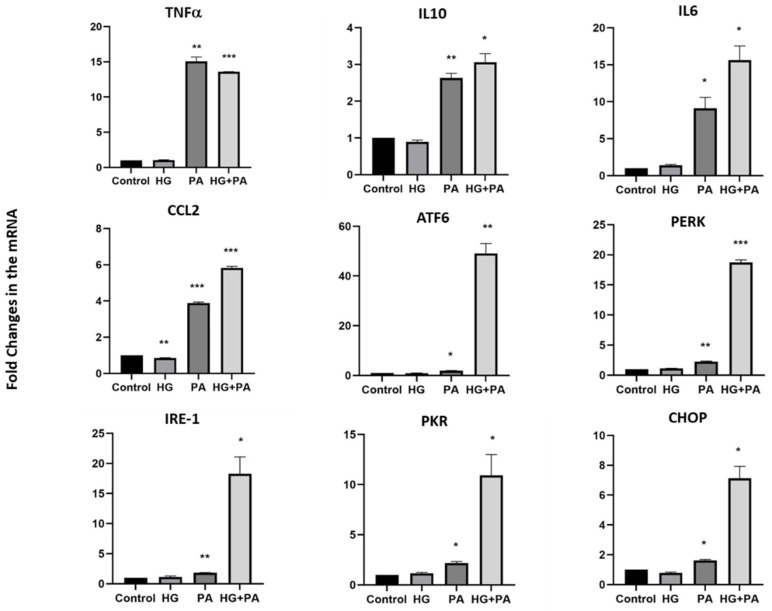
Gene expression levels of inflammatory and ER stress markers in THP1 cells treated for twenty-four hours. Gene expression levels of inflammatory markers (TNFα, IL10, IL6, CCL2) and ER stress markers (ATF6, PERK, IRE1, PKR and CHOP) were measured by quantitative real-time PCR in THP1 cells treated with high glucose (25 mM), palmitate (200 µM) and their combination for 24 h (*n* = 3 for each condition). Data are normalized to internal GAPDH and presented as fold-change compared with Control data. One-way ANOVA with repeated measurements followed by Sidak’s post hoc test were used to determine the significance of differences in means between the groups (* *p* < 0.05, ** *p* < 0.01, *** *p* < 0.001).

To elucidate whether the impact of metabolic stress on neuropeptide expression was related to endoplasmic reticulum (ER) stress and inflammatory markers commonly increased during obesity, THP1 cells were treated with either HG, PA, or their combination for 4 and 24 h, and the mRNA levels of selected genes were measured. After a 4 h treatment, an increase in expression was observed for most genes examined, especially in response to the HG and PA combination (Figure 4). However, a more pronounced increase in expression of inflammatory markers (tumor necrosis factor (TNFα), interleukin-10 (IL10), interleukin-6 (IL6), and chemokine C-C motif ligand 2 (CCL2)) as well as ER stress markers ((activating transcription factor 6 (ATF6), protein kinase R (PKR)-like endoplasmic reticulum kinase (PERK), Inositol-requiring enzyme 1 (IRE1), protein kinase RNA-activated (PKR), and C/EBP homologous protein (CHOP)) was observed in response to PA treatment for 24 h (*p* < 0.001, Figure 5). However, only marginal effects were observed on these markers in the presence of HG alone, whereas increased expression of the inflammatory markers, IL10, IL6, and CCL2, were observed in response to combined exposure of THP1 cells to HG and PA. This effect was also further exacerbated for the ER stress markers, ATF6, PERK, IRE1, PKR, and CHOP, in response to the combined treatment (Figure 5).

## 4. Discussion

This is the first study to report impaired neuropeptides CRF family transcripts in PBMCs of children with increased BMI. The main findings were: (1) significant decrease in UCN1, UCN3, and CRH expression with increased BMI in children; (2) at the transcript level, UCN3 strongly correlated with UCN1, UCN2 and CRH; (3) exposure of THP1 cells to high glucose, palmitate, or their combination for a short time (4 h) decreased UCN3 and CRH, whereas chronic exposure for 24 h increased UCN2, UCN3, and CRH expression with concomitant exacerbation of inflammatory and ER stress markers, suggesting crosstalk between CRF family neuropeptides and cellular stress.

CRH and the UCNs are expressed at the cellular level in immune cells, such as lymphocytes, macrophages, fibroblasts, and mast cells, as well as immunological tissues including the spleen and thymus [4]. Corticosterone is the primary hormone of the pituitary adrenocortical axis responsible for metabolism, stress, and adaptation. CRFR2 agonists modulate plasma corticosterone concentrations in a dose-dependent manner because of the synthesis and release of CRH, whereas intermediate doses decrease and higher doses enhance or do not change plasma corticosterone levels [4,25]. Increased glucocorticoids also induce the expression of UCN1, UCN2, and UCN3 [27,28,29]. Furthermore, CRFR2 and UCN3 are concomitantly present in the hypothalamus, lateral septum, and medial amygdala [30]. Altogether, these observations support a key role of CRF neuropeptides in modulating behavioral and hormonal responses to stress. Moreover, increased glucocorticoid and activation of cortisol from cortisone exacerbates metabolic stress, including obesity [31], suppresses insulin secretion, induces insulin resistance, and reduces peripheral glucose uptake [32]. In the present study, we observed a decrease in CRH, UCN1, and UCN3 levels in PBMCs with increased body weight, indicating a possible feedback mechanism to reduce the increase in glucocorticoids and hence, curb progression of pediatric obesity.

Nonetheless, while a significant decrease in mRNA levels of UCN1, UCN3, and CRH was observed in both overweight and obese children as compared to normal-weight controls, the plasma levels of these neuropeptides displayed an opposite trend (Table 1). Instead, UCN2 and UCN3 plasma levels significantly increased in overweight children as compared to normal-weight controls. In obese children, however, the plasma levels of UCN2, UCN3, and CRH significantly decreased to comparable levels with normal-weight children (Table 1). These opposite trends are suggestive of some compensatory mechanisms yet to be elucidated. The contribution of other organs to the circulating levels of these neuropeptides might be another source of these opposite trends. In a previous study, we also observed opposite trends in the UCN3 levels as measured in plasma and adipose tissue from human normal weight and obese adults [17].

UCN2 has been associated with a preference for high-fat food, whereas high hypothalamus UCN2 expression positively correlated with a preference for high-fat food in rats [33]. However, when centrally administered, UCN2 decreased high-fat diet intake in lean and diet-induced obese rats [34]. Children with a high consumption of fatty food exhibited low UCN2 expression levels in blood cells compared with a low or intermediate consumption of fatty food [24]. However, in our study, we cannot assess the possible impact of diet on UCNs expression levels, as we did not collect any information on children’s nutritional aspects. Nevertheless, we did not observe any significant changes in UCN2 transcript levels in our cohort with increased BMI in contrast to UCN1, UCN3, and CRH. This may result from a compensatory effect between UCN2 and UCN3 expression. These observations were further supported by our results using THP1 cells treated for a short time with high glucose and palmitate to imitate early-stage metabolic stress, such as obesity in children.

Several converging studies suggest the direct and local pro-inflammatory role of UCN1 and CRH as a result of environmental insult. For example, in rheumatoid arthritis, both UCN1 and CRH expression levels were increased in intestinal macrophages of ulcerative colitis patients, suggesting a pro-inflammatory role [35]. In correlation to the severity of inflammation in rheumatoid arthritis and osteoarthritis, UCN1 expression was markedly increased in patient leukocytes and macrophages [36]. The pro-inflammatory role of UCN1 is further supported by stimulation of IL1β and IL6 production in PBMCs through CRFR1 [37]. In the present study, the inflammatory marker, TNFα, was markedly increased in overweight children along with a significant reduction in UCN1 expression, especially in children with obesity. Thus, this opposite behavior of UCN1 could be a protective mechanism to mitigate the increased inflammatory response in obesity. UCN2 also exerts anti-inflammatory actions through a reduction of TNFα and IL6 expression in LPS-induced macrophages [38]. Tsatsanis demonstrated the anti-inflammatory effects of UCN2 in macrophage apoptosis and IL6 production [39]. Although we did not observe statistically significant differences in UCN2 transcript levels following a 4 h PA treatment, there was a significant increase in UCN2 expression along with elevated inflammatory and ER stress markers in THP1 cells treated for 24 h. The increased UCN2 levels may be part of a mechanism to curb inflammatory responses and maintain homeostasis as reported in previous studies [40].

Decreased CRH and UCN3 levels were observed in THP1 cells following short-term treatment with high glucose or palmitate, whereas spexin mRNA was decreased in response to combined exposure to high glucose and palmitate. However, during long term exposure, there was a significant increase in CRH, UCN2, and UCN3 expression in response to palmitate alone or in response to combined treatment with high glucose and palmitate. This may be a reaction to the associated increase in mRNA expression of inflammatory and ER stress markers observed with chronic fatty acid and combined treatment in THP1 cells. In contrast, the increase in stress markers was not observed with longer high glucose treatment, which suggests that high glucose conditions do not induce a stress response; hence, CRH and other neuropeptides showed a decrease or no change in expression, respectively. Consistent with these observations, metabolic stress levels experienced by children at early stages of obesity and during growth may be mitigated by the stress-related physiological response compared with adult and elderly humans, in which chronic exposure to cellular stress and a continued BMI increase leads to a chronic stress response similar to that observed with THP1 treated for longer times. Of note, the UCN3 plasma levels were significantly increased in overweight children but blunted in those with obesity. In our previous study of non-diabetic overweight adults, we reported decreased levels of circulating UCN3, which is similar to the UCN3 pattern that we demonstrate here in PBMCs [17]. However, the circulating UCN3 levels subsequently increased in overweight diabetic adults [17]. These evolving patterns with age and chronicity of metabolic stress may shed light on the shift between protective to pro-inflammatory roles for CRF neuropeptides in obesity in the pediatric population as they progress to adulthood.

Another neuropeptide with similar function to the CRH family is spexin, which is reported to be involved in energy metabolism and weight regulation [21]. We previously reported attenuated levels of plasma spexin with obesity and diabetes in adults; however, regular physical activity was able to normalize these levels [23]. Nevertheless, we did not observe any significant changes in children with increased BMI compared with normal weight children in the present study. Consistent with this result, Hodges et al. [22] reported that spexin had no correlation with body composition parameters, and there was no change in spexin levels with obesity or diabetes in adolescent patients. This indicates that spexin may not play a role as a metabolic regulator in adolescents; thus, it may have different effects based on age.

To the best of our knowledge, this is the first study to report impaired expression of specific neuropeptides in PBMCs of overweight or obese children in crosstalk with cellular and ER stress responses and supported by a cellular work. Differential dysregulation in the levels of CRF neuropeptides in PBMCs is indicative of an early adaptive response to metabolic disturbances and suggests a possible compensatory mechanism to blunt the increase in body weight. However, our study had some limitations. First, the cross-sectional study design did not allow us to determine whether the dysregulated neuropeptide levels contributed to the development of obesity. Second, the low number of participants limited the power of correlation analyses between neuropeptide levels and other clinical parameters. Third, our results may be altered, in part, by age and puberty-related physiological changes. Furthermore, no data regarding family history, diet, or physical activity of the children were collected, which were beyond the scope of this study. In addition, the measured expression of the neuropeptides in PBMCs may not reflect the levels in other tissues and organs. Finally, further in vitro and in vivo studies are needed to prove clinical significance and to elucidate the mechanisms and crosstalk between CRF neuropeptides and the cellular response to mitigate metabolic stress.

## Figures and Tables

**Table 1:** Characteristics of the study population.

Characteristics	Mean ± SD			*p* Value			Overall *p*
	Normal weight, NW (*n* = 8)	Overweight, OW (*n* = 10)	Obese (OB) (*n* = 22)	NW vs. OW	NW vs. OB	OW vs. OB	All Children
Physical and clinical characteristics							
Sex (M/F)	5/3	3/7	14/8	0.188	0.956	0.082	0.196
Age (years)	10.5 ± 4.3	12.8 ± 3.4	11.3 ± 2.1	0.260	0.788	0.429	0.265
Weight (Kg)	38.2 ± 18.1	58.5 ± 14.6	83.6 ± 30.9	0.230	<0.001	0.037	<0.001
Height (m)	1.44 ± 0.25	1.54 ± 0.12	1.52 ± 0.17	0.482	0.511	0.969	0.460
BMI (Kg/m^2^)	17.16 ± 2.59	24.22 ± 2.97	35.05 ± 6.95	0.029	<0.001	<0.001	<0.001
Percentile ^#^	41.0 ± 18.8	91.8 ± 1.8	98.8 ± 0.6	<0.001	<0.001	0.078	<0.001
Body fat (%)	6.1 ± 3.6	16.7 ± 6.8	32.9 ± 15.3	0.003	<0.001	0.005	<0.001
Metabolic and Hormonal markers							
Cholesterol (mmol/L)	4.09 ± 0.62	4.57 ± 0.51	4.11 ± 0.96	0.451	0.997	0.357	0.344
HDL (mmol/L)	1.43 ± 0.31	1.34 ± 0.15	1.10 ± 0.28	0.748	0.013	0.074	0.008
LDL (mmol/L)	2.33 ± 0.59	2.82 ± 0.44	2.57 ± 0.82	0.323	0.692	0.639	0.355
TGL (mmol/L)	0.71 ± 0.36	0.99 ± 0.51	1.00 ± 0.45	0.410	0.295	0.999	0.297
GLU (mmol/L)	4.5 ± 0.4	5.6 ± 2.0	5.0 ± 0.4	0.120	0.633	0.326	0.132
HbA1c (%)	5.2 ± 0.3	5.7 ± 2.0	5.2 ± 0.7	0.670	0.998	0.525	0.528
Insulin (mIU/L)	3.6 ± 1.8	9.4 ± 4.4	17.15 ± 6.8	0.003	0.016	0.08	0.012
HOMA IR	0.74 ± 0.39	2.59 ± 2.34	5.54 ± 5.43	0.043	0.001	0.010	0.001
Adiponectin (µg/mL)	9.8 ± 1.1	8.6 ± 1.3	7.6 ± 2.7	0.496	0.057	0.473	0.065
TNFα (ng/mL)	1.21 ± 0.59	31.79 ± 9.40	25.86 ± 34.60	0.046	0.073	0.826	0.344
Other markers							
Leptin (ng/mL)	7.78 ± 2.88	13.12 ± 6.67	11.17 ± 4.04	0.053	0.197	0.521	0.041
C-pep (ng/mL)	366 ± 219	187 ± 75	112 ± 41	0.006	<0.001	0.221	0.064
Glucagon (ng/mL)	4.04 ± 1.81	1.62 ± 0.51	2.38 ± 1.14	<0.001	0.005	0.236	<0.001
NGAL (ng/mL)	24.71 ± 8.59	36.57 ± 17.87	24.31 ± 5.02	0.058	0.996	0.013	<0.001
RBP4 (µg/mL)	19.45 ± 2.92	54.97 ± 19.74	19.10 ± 5.75	<0.001	0.997	<0.001	0.013
sICAM-1 (ng/mL)	132 ± 31	284 ± 233	157 ± 83	0.057	0.890	0.057	<0.001
ZAG (µg/mL)	1.60 ± 0.22	5.02 ± 0.71	1.70 ± 0.37	<0.001	0.847	<0.001	0.037
Resistin (ng/mL)	5.16 ± 2.98	4.76 ± 1.46	6.75 ± 2.92	0.950	0.337	0.166	<0.001
Circulating CRH (pg/mL)	76.80 ± 103.89	136.34 ± 105.24	23.38 ± 44.55	0.242	0.222	0.001	0.132
Circulating UCN1 (pg/mL)	43.38 ± 42.12	15.35 ± 27.67	32.30 ± 32.41	0.195	0.704	0.388	0.002
Circulating UCN2 (ng/mL)	1297 ± 362	1832 ± 400	1397 ± 374	0.014	0.800	0.013	0.206
Circulating UCN3 (ng/mL)	2.94 ± 0.99	33.28 ± 37.81	2.33 ± 0.32	0.006	0.997	0.001	0.007
Circulating Spexin (ng/mL)	14.67 ± 1.59	13.14 ± 2.13	18.04 ± 4.38	0.648	0.071	0.004	0.001

^#^: Only the percentile between 95 and 99 were included for the obese children. Data are presented as the mean ± SD. ALT (alanine transaminase), AST (aspartate transaminase), C-pep (connecting peptide), GGT (gamma-glutamyltransferase), HbA1c (hemoglobin A1c), HDL (high-density lipoprotein), HOMA IR (homeostatic model assessment for insulin resistance), LDL (low-density lipoprotein), NGAL (neutrophil gelatinase-associated lipocalin), TGL (triglyceride), RBP4 (retinol binding protein 4, sICAM-1 (soluble intercellular adhesion molecule-1, ZAG (zinc alpha 2-glycoprotein). One-way ANOVA followed by Bonferroni’s post hoc test.

**Table 2:** Spearman correlation ranking of neuropeptides with study characteristics.

All Children	FC CRH	FC UCN1	FC UCN2	FC UCN3	FC SPX
Sex (M/F)	−0.045	−0.023	0.063	−0.089	−0.039
Age (years)	−0.323	−0.169	−0.186	−0.069	−0.186
Weight (Kg)	−0.091	−0.352 *	0.026	−0.129	0.093
Height (m)	−0.030	−0.240	−0.030	−0.033	−0.085
BMI (Kg/m *)	−0.141	−0.362 *	−0.295	−0.308	0.291
Percentiles	−0.132	−0.351 *	−0.143	−0.234	0.196
Body fat (%)	−0.019	−0.423 *	0.087	−0.051	0.059
Cholesterol (mmol/L)	−0.101	−0.427 **	0.031	−0.164	0.120
HDL (mmol/L)	0.145	0.071	0.011	0.094	0.125
LDL (mmol/L)	0.058	0.261	−0.130	0.140	−0.232
TGL (mmol/L)	0.144	−0.034	0.058	0.037	0.252
GLU (mmol/L)	−0.043	0.047	0.127	−0.039	0.208
HBA1C (%)	−0.203	−0.039	0.028	−0.230	−0.032
Insulin (mIU/L)	−0.040	−0.047	0.182	0.075	−0.244
HOMA IR	−0.239	−0.333	−0.256	−0.317	0.048
Adiponectin (µg/mL)	0.086	0.254	−0.139	0.003	−0.019
TNFα (ng/mL)	−0.387 *	−0.050	−0.208	−0.388 *	0.083
Leptin (ng/mL)	−0.197	−0.005	0.176	−0.212	0.289
C−pep (pg/mL)	0.069	0.257	0.193	0.219	−0.121
glucagon Final (ng/mL)	0.269	0.119	−0.025	0.221	−0.081
NGAL (ng/mL)	−0.220	0.256	0.066	−0.343 *	0.206
RBP4 (µg/mL)	−0.415 *	−0.027	−0.056	−0.328	−0.175
sICAM−1 (ng/mL)	−0.323	0.067	0.174	−0.122	−0.136
ZAG (µg/mL)	−0.428 *	−0.082	0.054	−0.224	−0.190
Resistin (ng/mL)	−0.068	−0.235	−0.078	−0.205	0.057
Circulating CRH (pg/mL)	−0.327	0.091	0.058	−0.081	−0.228
Circulating UCN1 (pg/mL)	0.097	−0.209	−0.121	−0.090	0.114
Circulating UCN2 (ng/mL)	−0.353 *	−0.274	−0.215	−0.209	−0.014
Circulating UCN3 (ng/mL)	0.050	0.298	0.545 **	0.331 *	−0.083
Circulating Spexin (ng/mL)	0.106	−0.304	−0.439 *	−0.263	0.307
FC CRH	−	0.293	0.663 **	0.668 **	0.258
FC UCN1	0.293	−	0.377	0.409 *	0.071
FC UCN2	0.663 **	0.377	−	0.887 **	0.163
FC UCN3	0.668 **	0.409 *	0.887 **	−	−0.056
FC SPX	0.258	0.071	0.163	−0.056	−

Data are presented as the R values. Significances of Spearman correlation are as follows: * *p* < 0.05, ** *p* < 0.01. ALT (alanine transaminase), AST (aspartate transaminase), C-pep (connecting peptide), GGT (gamma-glutamyltransferase), HbA1c (hemoglobin A1c), HDL (high-density lipoprotein), HOMA IR (homeostatic model assessment for insulin resistance), LDL (low-density lipoprotein), NGAL (neutrophil gelatinase-associated lipocalin), TGL (triglyceride), RBP4 (retinol binding protein 4, sICAM-1 (soluble intercellular adhesion molecule-1, ZAG (zinc alpha 2-glycoprotein).

## Data Availability

All generated data and resources are reported in this manuscript and there are no other data to be provided.

## Supplementary Materials

The following supporting information can be downloaded at: https://www.mdpi.com/article/10.3390/nu14030429/s1, Table S1. List of human primers; Figure S1. Protein expression levels in THP1 cells treated for 24 h (original Western blots).
