# Supplementary material for: Urocortin Neuropeptide Levels Are Impaired in the PBMCs of Overweight Children
Source: Nutrients. 2022 Jan 18;14(3):429. doi: 10.3390/nu14030429 (PMC8839374; doi:10.3390/nu14030429)
Supplement: Supplementary file 1 [file nutrients-14-00429-s001.zip › nutrients-1535163-supplementary.pdf]

Table S1. List of human primers.

**Primers**	***Catalog number or sequence***
UCN1	*Hs01849155_s1* (Applied Biosystem, Foster City, CA, USA)
UCN2	*Hs00264218_s1* (Applied Biosystem, Foster City, CA, USA)
UCN3	*Hs00846499_s1* (Applied Biosystem, Foster City, CA, USA)
CRH	*Hs01921237_s1* (Applied Biosystem, Foster City, CA, USA)
Spexin	Hs00228976_m1 (Applied Biosystem, Foster City, CA, USA)
TNF-α	*Hs01113624_g1* (Applied Biosystem, Foster City, CA, USA)
IL-10	*Hs00961622_m1* (Applied Biosystem, Foster City, CA, USA)
IL-6	*Hs00985639_m1* (Applied Biosystem, Foster City, CA, USA)
CCL-2	*Hs00234140_m1* (Applied Biosystem, Foster City, CA, USA)
ATF-6	*5′-ACCCACTAAAGGCCAGACG-3′*<br>*5′-CCACGTGATTAGGGAGCTGT-3′*
PERK	*5′-ATGATCATTCCTTCCCTGGAT-3′*<br>*5′-AGTCAGAGATTTTCCTCCAACC-3′*
IRE-1	*5′-GCAAGAGGACAGGCTCAATC-3′*<br>*5′-ACGTCCTTTGAGCAGAATGC-3′*
PKR	*5′-TCGCTGGTATCACTCGTCTG-3′*<br>*5′-GATTCTGAAGACCGCCAGAG-3′*
CHOP	*5′-TGA GGA GAG AGT TAG GTA ATT CC-3′*<br>*5′-TTT AAA ACA GGT CAT TCC TCT GC-3′*

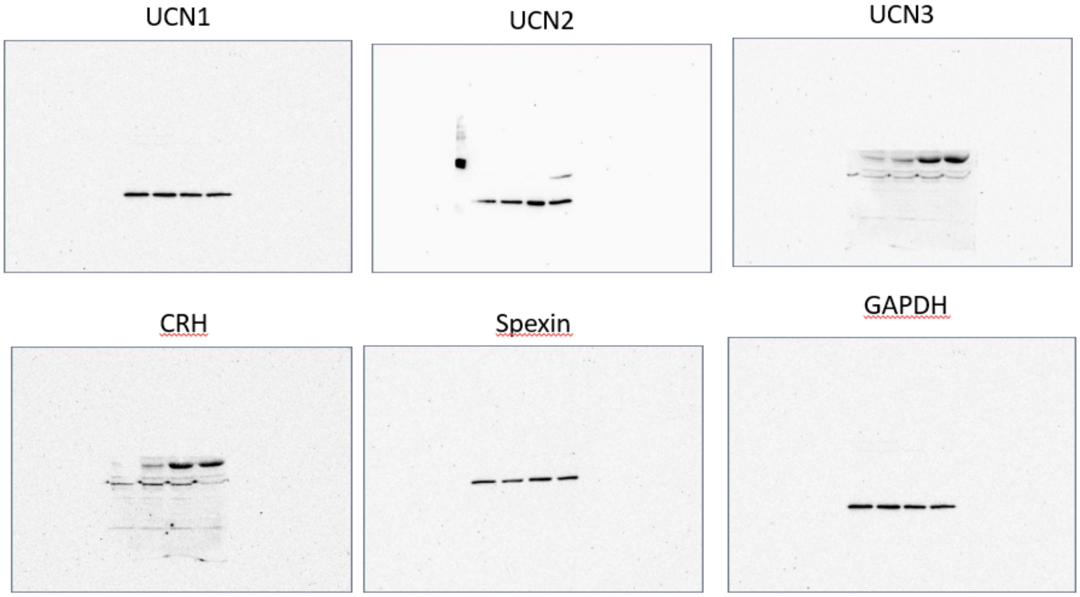

Supplementary Figure S1. Protein expression levels in THP1 cells treated for 24h (original Western blots).
